# Supplementary material for: Development of a Novel LC-MS/MS Multi-Method for the Determination of Regulated and Emerging Food Contaminants Including Tenuazonic Acid, a Chromatographically Challenging Alternaria Toxin
Source: Molecules. 2023 Feb 2;28(3):1468. doi: 10.3390/molecules28031468 (PMC9921091; doi:10.3390/molecules28031468)
Supplement: Supplementary file 1 [file molecules-28-01468-s001.zip › molecules-2197703-supplementary.pdf]

molecules

MDPI

Article

Development of a novel LC-MS/MS multi-method for the determination of regulated and emerging food contaminants including tenuazonic acid, a chromatographically challenging *Alternaria* toxin

Ádám Tölgyesi[1,*], Attila Cseh[1], Andrea Simon[1] and Virender K. Sharma[2,*]

1 Mertcontrol Hungary Ltd, Szabadság út 13., 2144 Kerepes, Hungary
2 Program for the Environment and Sustainability, Department of Environmental and Occupational Health, School of Public Health, Texas A&M University, 212 Adriance Lab Road, 1266 TAMU, College Station, TX 77843, USA
* Correspondence: atolgyesi@mertcontrol.com; (Á.T.); vsharma@tamu.edu; VKS

Table S1: Quality control and proficiency test materials.

QC name	Code	Origin	Matrix	Compound group	Compounds with assigned value ± standard deviation, expressed in μg/kg
EURL2017	EA047	JRC (Geel, Belgium)	Rye	Ergot alkaloid	Ergocornine/inine SUM 295±65; α-Ergocryptine/inine SUM 231±51; Ergocrystine/inine SUM 752±165; Ergometrine/inine SUM 116±26; Ergosine/inine SUM 242±53; Ergotamine/inine SUM 695±153
FAPAS	22180	Fera (Sand Hutton, UK)	Rye	Ergot alkaloid	Ergocornine 32.4±7.2; Ergocorninine 12.4±2.7; α-Ergocryptinine 19.5±4.3; Ergocrystine 107±23; Ergocrystinine 36.4±8; Ergometrin 25.9±5.7; Ergometrinine 4.2±0.95; Ergosine 22.2±4.9; Ergotamin 50.2±11.1; Ergotaminine 13.6±2.9; Total Ergot Alkaloides 419±76;
EURL2017	A004	JRC (Geel, Belgium)	Wheat	DON	DON 551±121
EURL2016	C257	JRC (Geel, Belgium)	Maize	Mycotoxin	DON 611±134<br>AFB1 10.6±2.3<br>FUMB1 768±170<br>FUMB2 224±49<br>ZON 162±36
EURL2016	O161	JRC (Geel, Belgium)	Oat	Mycotoxin	HT2 150±33<br>T2 70.3±16
EURL2016	E087	JRC (Geel, Belgium)	Cereal	Tropan alkaloids	Atropine 7.44±1.52; Scopolamine 1.03±0.22;
EURL2016	C029	JRC (Geel, Belgium)	Cereal	Tropan alkaloids	Atropine 1.16±0.25; Scopolamine 0.183±0.081;
Fapas	22179	Fera (Sand Hutton, UK)	Cereal	Tropan alkaloids	Atropine 9.8±2,16; Scopolamine 3.88±0.85;

QC 2018	B56	JRC (Geel, Belgium)	Wheat	Alternaria	AME 1.17±0.25;<br>AOH 2.1±0.46;<br>TEA 297±60;
QC 2018	T15	JRC (Geel, Belgium)	Wheat	Alternaria	AME 5.06±1.11;<br>AOH 5.11±1.12;<br>TEA 52±11;
QC 2018	X06	JRC (Geel, Belgium)	Sunflower seeds	Alternaria	AME 2.1±0.45;<br>AOH 2.06±0.44;<br>TEA 146±32;
Romer QC	10006460	RomerLabs (Tulln, Austria)	Wheat	Don, Zon, Ochra	DON 825±124;<br>ZON 34.9±5.95;<br>OTA 10±2.1;
Romer QC	10003613	RomerLabs (Tulln, Austria)	Maize	Aflatoxins	AFB1 9.5±2.1;<br>AFB2 2.1±0.4;
Trilogy	TQC-MMF11-100	R-Biopharm (Budapest, Hungary)	Maize	Multi-Toxin	DON 1900±200<br>AFB1 18.6±4.1<br>AFB2 1.3±0.28<br>Total Aflatoxins 19.9±4.4<br>FUMB1 1400±150<br>FUMB2 400±35<br>FUMB3 100±15<br>Total Fumonisins 1900±200<br>HT2 127±12.3<br>T2 94.8±7.95<br>OTA 18.5±3.1<br>ZON 359.7±40.1
Gafta PT	2022-M2	Gafta (London, UK)	Maize	Aflatoxins	AFB1 1.60±0.35<br>AFB2 0.50±0.11<br>AFG1 1.50±0.33<br>AFG2 0.5±0.11<br>Total Aflatoxins 3.73±0.82
Gafta PT	2022-M1	Gafta (London, UK)	Wheat	T2, HT2	HT2 7.3±3.2<br>T2 43.8±19.3
Romer PT	M21161DZO	RomerLabs (Tulln, Austria)	Wheat	Don, Zon, Ochra	DON 2841±255;<br>OTA 30.7±7.75;<br>ZON 177±38.9;

Romer PT	M22411AF	RomerLabs (Tulln, Austria)	Maize	Aflatoxins, fumonisins	AFB1 8.79±1.93<br>AFB2 0.63±0.14<br>AFG1 0.49±0.11<br>Total Aflatoxins 9.72±2.14<br>FUMB1 1425±185<br>FUMB2 387±75<br>FUMB3 168±35.5<br>Total Fumonisins 1911±230
Romer PT	M22161DZO	RomerLabs (Tulln, Austria)	Wheat	Don, Zon, Ochra	DON 1826±264;<br>OTA 25.9±5.6;<br>ZON 545±86.5;
FAPAS	T09133QC	Fera (Sand Hutton, UK)	Kidney beans	Pesticides	Boscalid 107±23;<br>Chlorpyrifos-ethyl 103±23;<br>Flufenoxuron 55±12;<br>Flusilazole 155±32<br>Isofenphos-methyl 77±17;<br>Isoprothiolane 156±33;<br>Methacrifos 119±27;<br>Pirimicarb 101±22;<br>Pyridaben 53±12;<br>Thiacloprid 85±18;
FAPAS	T09140QC	Fera (Sand Hutton, UK)	Wheat fluor	Pesticides	Chlorpropham 58.9±13<br>Dicrotophos 33.2±7.3<br>Dimetoate 32.1±7.1<br>Oxadiazon 77.8±17.1<br>Paclobutrazole 98.3±21.6<br>Permethrin 64.7±14.3<br>Pirimiphos-methyl 104±23<br>Prochloraz 153±32.5<br>Tebuconazole 87.3±19.2
PT, Chlorpyrifos-ethyl	6	Bálint Analitika Ltd. (Budapest, Hungary)	Wheat	Chlorpyrifos-ethyl	Chlorpyrifos-ethyl 33.0±7.26
PT, Chlorpyrifos-ethyl	8	Mertcontrol-HL Ltd. (Debrecen, Hungary)	Wheat	Chlorpyrifos-ethyl	Chlorpyrifos-ethyl 16.9±3.72

Table S2: The scheduled MRM ion transitions of the tested compounds.

Compound Name	Precursor Ion (m/z)	Product Ion (m/z)	Retention Time (min)	Fragmentor (V)	Collision Energy (V)	Polarity
Acephate	183.9	143	2.8	65	2	Positive
Acephate	183.9	125	2.8	65	17	Positive
Acetamiprid	223	126	6.4	83	22	Positive
Acetamiprid	223	90	6.4	83	35	Positive
Afla B1	313	285	8.2	155	22	Positive
Afla B1	313	241	8.2	155	38	Positive
Afla B1-^{13}C17	330	301	8.2	155	21	Positive
Afla B2	315	287	7.8	155	24	Positive
Afla B2	315	259	7.8	155	28	Positive
Afla B2-^{13}C17	332	303	7.8	155	21	Positive
Afla G1	329	311	7.5	140	22	Positive
Afla G1	329	243	7.5	140	24	Positive
Afla G1-^{13}C17	346	257	7.5	140	24	Positive
Afla G2	331	313	7.1	155	23	Positive
Afla G2	331	245	7.1	155	28	Positive
Afla G2-^{13}C17	348	330	7.1	155	23	Positive
Alanycarb	400	238	12.8	140	4	Positive
Alanycarb	400	91	12.8	140	50	Positive
Aldicarb	208	116	7.4	65	0	Positive
Aldicarb	208	89	7.4	65	12	Positive
Aldicarb fragment	116	89	7.4	65	4	Positive
Aldicarb fragment	116	70	7.4	65	4	Positive
AME	271	256	12.03	110	22	Negative
AME	271	228	12.03	110	28	Negative
AME-d3	274	256	12.03	110	22	Negative
AME-d3	274	228	12.03	110	28	Negative
Amidosulfuron	370	261	5.7	100	12	Positive
Amidosulfuron	370	218	5.7	100	22	Positive
Aminocarb	209	152	8.5	105	12	Positive
Aminocarb	209	137	8.5	105	24	Positive
AOH	257	215	9.3	110	26	Negative
AOH	257	213	9.3	110	26	Negative
AOH	257	147	9.3	110	34	Negative
AOH-d2	259	216	9.3	110	24	Negative
AOH-d2	259	215	9.3	110	24	Negative

AOH-d2	259	214	9.3	110	24	Negative
AOH-d2	259	213	9.3	110	24	Negative
Atropine	290	124	6.2	135	26	Positive
Atropine	290	93	6.2	135	35	Positive
Atropine-d5	295	124	6.2	135	26	Positive
Avermectin B1a	890.5	567	16.1	120	5	Positive
Avermectin B1a	890.5	305	16.1	120	24	Positive
Azaconazole	300	231	10.1	140	16	Positive
Azaconazole	300	159	10.1	140	32	Positive
Azamethiphos	325	183	8.1	120	12	Positive
Azamethiphos	325	112	8.1	120	37	Positive
Azinphos-ethyl	346	137	12	120	32	Positive
Azinphos-ethyl	346	132	12	120	31	Positive
Azinphos-methyl	318	261	10.4	60	0	Positive
Azinphos-methyl	318	132	10.4	60	8	Positive
Azoxystrobin	404	372	10.8	110	12	Positive
Azoxystrobin	404	344	10.8	110	24	Positive
Beflubutamid	356	162	12.7	145	24	Positive
Beflubutamid	356	91	12.7	145	28	Positive
Benalaxyl	326	294	12.9	100	5	Positive
Benalaxyl	326	148	12.9	100	17	Positive
Benfuracarb	411	252	14.2	95	12	Positive
Benfuracarb	411	195	14.2	95	22	Positive
Benzoximate	364	199	13.4	83	4	Positive
Benzoximate	364	105	13.4	83	22	Positive
Bifenazate	301	198	11.8	95	5	Positive
Bifenazate	301	170	11.8	95	17	Positive
Bifenthrin	440	181	16.5	110	5	Positive
Bifenthrin	440	166	16.5	110	22	Positive
Bispyribac	431	413	9.6	115	12	Positive
Bispyribac	431	275	9.6	115	5	Positive
Bitertanol	338	269	13.3	65	0	Positive
Bitertanol	338	70	13.3	65	0	Positive
Bosclid	343	307	11.2	145	12	Positive
Bosclid	343	271	11.2	145	28	Positive
Bromuconazole	378	159	11.7	115	35	Positive
Bromuconazole	378	70	11.7	115	22	Positive
Bupirimate	317	166	12.5	125	22	Positive
Bupirimate	317	108	12.5	125	24	Positive
Buprofezin	306	201	14.5	105	5	Positive
Buprofezin	306	116	14.5	105	12	Positive

Butocarboxim1	208	116	5.5	65	12	Positive
Butocarboxim1	208	75	5.5	65	22	Positive
Butocarboxim2	213	156	7.3	65	5	Positive
Butocarboxim2	213	75	7.3	65	12	Positive
Carbaryl	202	145	8.8	65	0	Positive
Carbaryl	202	127	8.8	65	24	Positive
Carbendazim	192	160	6.9	105	17	Positive
Carbendazim	192	132	6.9	105	28	Positive
Carbofuran	222	165	8.4	83	5	Positive
Carbofuran	222	123	8.4	83	22	Positive
Carbosulfan	381	160	16.2	105	8	Positive
Carbosulfan	381	118	16.2	105	16	Positive
Carboxin	236	143	8.8	105	12	Positive
Carboxin	236	93	8.8	105	37	Positive
Carfentrazone-ethyl	412	366	12.7	140	17	Positive
Carfentrazone-ethyl	412	346	12.7	140	22	Positive
Chlorantraniliprole	484	453	10.4	105	17	Positive
Chlorantraniliprole	484	286	10.4	105	12	Positive
Chlorfenvinphos	359	170	13	105	37	Positive
Chlorfenvinphos	359	155	13	105	8	Positive
Chloridazon	222	104	6.5	140	24	Positive
Chloridazon	222	77	6.5	140	35	Positive
Chlorotoluron	213	140	9.4	120	22	Positive
Chlorotoluron	213	72	9.4	120	22	Positive
Chloroxuron	291	164	11.7	140	12	Positive
Chloroxuron	291	72	11.7	140	22	Positive
Chlorpyriphos-ethyl	352	200	14.9	110	17	Positive
Chlorpyriphos-ethyl	350	198	14.9	110	17	Positive
Chlorpyriphos-ethyl -d10	360	199	14.8	110	17	Positive
Chlorpyriphos-ethyl -d10	360	163	14.8	110	21	Positive
Chlorpyriphos-ethyl -d10	360	99	14.8	110	17	Positive
Chlorpyriphos-methyl	322	290	13.7	110	12	Positive
Chlorpyriphos-methyl	322	125	13.7	110	24	Positive
Chlorsulfuron	358	167	6.3	120	12	Positive
Chlorsulfuron	358	141	6.3	120	17	Positive
Clethodim	360	268	9.2	110	12	Positive

Clethodim	360	164	9.2	110	17	Positive
Clethodim2	360	268	10.1	110	12	Positive
Clethodim2	360	164	10.1	110	17	Positive
Clofentezin	303	138	13.4	110	12	Positive
Clofentezin	303	102	13.4	110	37	Positive
Clomazone	240	125	10.5	65	17	Positive
Clomazone	240	89	10.5	65	45	Positive
Coumaphos	363	307	13	120	16	Positive
Coumaphos	363	227	13	120	28	Positive
Cyazofamid	325	261	12.2	100	5	Positive
Cyazofamid	325	108	12.2	100	12	Positive
Cycloate	216	82.9	13.7	100	12	Positive
Cycloate	216	55	13.7	100	36	Positive
Cycluron	199	89	10	120	12	Positive
Cycluron	199	72	10	120	28	Positive
Cymiazol	219	171	11.8	95	24	Positive
Cymiazol	219	144	11.8	95	35	Positive
Cymoxanil	199	128	6.8	50	0	Positive
Cymoxanil	199	111	6.8	50	17	Positive
Cypermethrin	433	208	15.5	120	17	Positive
Cypermethrin	433	191	15.5	120	12	Positive
Cypermethrin	433	163	15.5	120	24	Positive
Cypermethrin	433	127	15.5	120	24	Positive
Cyproconazole	292	125	11.8	110	35	Positive
Cyproconazole	292	70	11.8	110	17	Positive
Cyprodinil	226	93	12.9	140	37	Positive
Cyprodinil	226	77	12.9	140	45	Positive
DEET	1924	119	9.8	110	16	Positive
DEET	1924	91	9.8	110	32	Positive
Deltamethrin	523	506	15.6	120	8	Positive
Deltamethrin	523	281	15.6	120	14	Positive
Desmedipham	318	182	10.3	83	5	Positive
Desmedipham	318	136	10.3	83	24	Positive
Diazinon	305	169	13	105	22	Positive
Diazinon	305	153	13	105	22	Positive
Dichlorvos	221	109	8.2	110	12	Positive
Dichlorvos	221	79	8.2	110	24	Positive
Diethofencarb	268	226	10.8	65	0	Positive
Diethofencarb	268	124	10.8	65	28	Positive
Difenoconazole	406	337	13.6	120	17	Positive
Difenoconazole	406	251	13.6	120	24	Positive

Diflubenzuron	311	158	12.4	83	12	Positive
Diflubenzuron	311	141	12.4	83	35	Positive
Diflufenican	395	266	13.8	140	24	Positive
Diflufenican	395	246	13.8	140	37	Positive
Dimethachlor	256	224	10.2	120	12	Positive
Dimethachlor	256	148	10.2	120	24	Positive
Dimethoate	230	199	6.4	65	0	Positive
Dimethoate	230	125	6.4	65	22	Positive
Dimethomorph	388	301	11.4	145	22	Positive
Dimethomorph	388	165	11.4	145	28	Positive
Dimoxystrobin	327	205	12.6	115	5	Positive
Dimoxystrobin	327	116	12.6	115	22	Positive
Diniconazole	326	159	13.5	110	28	Positive
Diniconazole	326	70	13.5	110	24	Positive
Dinotefuran	203	129	4.6	183	8	Positive
Dinotefuran	203	114	4.6	183	12	Positive
Dioxacarb	224	167	6.4	83	12	Positive
Dioxacarb	224	123	6.4	83	12	Positive
Disulfoton	275	89	14.3	140	5	Positive
Disulfoton	275	61	14.3	140	37	Positive
Diuron	235	72	10	110	22	Positive
Diuron	233	72	10	110	22	Positive
DON	297	249	5	110	4	Positive
DON	297	203	5	110	12	Positive
DON-^{13}C15	312	263	5	110	4	Positive
Epoxyconazol	330	121	12.1	110	22	Positive
Epoxyconazol	330	101	12.1	110	45	Positive
Ergocornine	562.4	305	12	147	24	Positive
Ergocornine	562.4	277	12	147	24	Positive
Ergocornine	562.4	223	12	147	36	Positive
Ergocorninine	562.4	305	13.3	147	24	Positive
Ergocorninine	562.4	277	13.3	147	24	Positive
Ergocorninine	562.4	223	13.3	147	36	Positive
Ergocristine	610.4	305	12.7	172	24	Positive
Ergocristine	610.4	268	12.7	172	24	Positive
Ergocristine	610.4	223	12.7	172	36	Positive
Ergocristinine	610.4	305	14	162	28	Positive
Ergocristinine	610.4	268	14	162	24	Positive
Ergocristinine	610.4	223	14	162	36	Positive
Ergocryptine	576.4	305	12.6	152	24	Positive
Ergocryptine	576.4	291	12.6	152	24	Positive

Ergocryptine	576.4	223	12.6	152	36	Positive
Ergocryptinine	576.4	305	13.8	201	28	Positive
Ergocryptinine	576.4	291	13.8	201	24	Positive
Ergocryptinine	576.4	223	13.8	201	36	Positive
Ergometrine	326	223	7.1	147	22	Positive
Ergometrine	326	208	7.1	147	36	Positive
Ergometrine	326	197	7.1	147	22	Positive
Ergometrinine	326	265	8.5	152	16	Positive
Ergometrinine	326	223	8.5	152	24	Positive
Ergometrinine	326	208	8.5	152	28	Positive
Ergosine	548.4	530	11.5	152	12	Positive
Ergosine	548.4	223	11.5	152	28	Positive
Ergosine	548.4	208	11.5	152	50	Positive
Ergosinine	548.4	530	12.7	196	12	Positive
Ergosinine	548.4	223	12.7	196	32	Positive
Ergosinine	548.4	208	12.7	196	50	Positive
Ergotamine	582.4	277	11.9	167	24	Positive
Ergotamine	582.4	223	11.9	167	32	Positive
Ergotamine	582.4	208	11.9	167	50	Positive
Ergotaminine	582.4	277	13.2	167	24	Positive
Ergotaminine	582.4	223	13.2	167	36	Positive
Ergotaminine	582.4	208	13.2	167	50	Positive
Ethidimuron	265	207.9	6.1	120	12	Positive
Ethidimuron	265	57	6.1	120	32	Positive
Ethion	385	199	14.7	95	4	Positive
Ethion	385	143	14.7	95	22	Positive
Ethirimol	210	140	10	145	22	Positive
Ethirimol	210	98	10	145	24	Positive
Ethofumesat	287	259	10.9	83	0	Positive
Ethofumesat	287	121	10.9	83	12	Positive
Ethoprophos	243	131	12.1	100	17	Positive
Ethoprophos	243	97	12.1	100	28	Positive
Ethoxyquin	218	174	12.2	120	28	Positive
Ethoxyquin	218	160	12.2	120	35	Positive
Etofenprox	394	177	16.5	110	12	Positive
Etofenprox	394	107	16.5	110	45	Positive
Etrimphos	293	265	12.9	120	22	Positive
Etrimphos	293	125	12.9	120	35	Positive
Famoxadone	392	331	13.1	85	0	Positive
Famoxadone	392	238	13.1	85	12	Positive
FB1	722.4	352.3	8.3	183	45	Positive

FB1	722.4	334.3	8.3	183	45	Positive
FB1-^{13}C34	756.5	356.4	8.3	183	45	Positive
FB2	706.5	336.3	10.9	183	45	Positive
FB2	706.5	318.3	10.9	183	45	Positive
FB2-^{13}C34	740.5	358.3	10.9	183	45	Positive
FB3	706.5	336.3	9.8	183	45	Positive
FB3	706.5	318.3	9.8	183	45	Positive
FB3-^{13}C34	740.5	358.3	9.8	183	45	Positive
Fenamidon	312	236	11.1	110	12	Positive
Fenamidon	312	92	11.1	110	24	Positive
Fenamiphos	304	217	12.4	110	22	Positive
Fenamiphos	304	202	12.4	110	35	Positive
Fenarimol	331	268	12	140	22	Positive
Fenarimol	331	81	12	140	28	Positive
Fenazaquin	307	161	15.8	105	12	Positive
Fenazaquin	307	57	15.8	105	24	Positive
Fenbuconazole	337	125	12.3	145	35	Positive
Fenbuconazole	337	70	12.3	145	17	Positive
Fenhexamid	302	97	11.9	140	22	Positive
Fenhexamid	302	55	11.9	140	37	Positive
Fenobucarb	208	152	10.7	65	5	Positive
Fenobucarb	208	95	10.7	65	12	Positive
Fenoxycarb	302	116	12.5	100	5	Positive
Fenoxycarb	302	88	12.5	100	17	Positive
Fenpropidin	274	147	14.1	120	28	Positive
Fenpropidin	274	86	14.1	120	24	Positive
Fenpyroximat	422	366	15.3	135	17	Positive
Fenpyroximat	422	135	15.3	135	28	Positive
Fenuron	165	76.9	6.2	183	32	Positive
Fenuron	165	72	6.2	183	16	Positive
Fipronil	435	330	12.5	65	12	Negative
Fipronil	435	250	12.5	65	28	Negative
Flazasulfuron	408	182	6.4	120	17	Positive
Flazasulfuron	408	139	6.4	120	37	Positive
Flonicamid	230	203	5.1	110	17	Positive
Flonicamid	230	174	5.1	110	17	Positive
Fluazinam	463	416	13.76	115	13	Negative
Fluazinam	463	398	13.76	115	9	Negative
Flubendiamide	681	273.7	12.7	120	12	Negative
Flubendiamide	681	254	12.7	120	24	Negative
Fludioxonil	247	169	11.1	95	32	Negative

Fludioxonil	247	126	11.1	95	32	Negative
Flufenacet	364	194	12.1	100	5	Positive
Flufenacet	364	152	12.1	100	17	Positive
Flufenoxuron	489	158	15.1	110	17	Positive
Flufenoxuron	489	141	15.1	110	45	Positive
Flumetsulam	326	262	5.3	135	17	Positive
Flumetsulam	326	129	5.3	135	22	Positive
Flumioxazin	355	327	10.4	110	22	Positive
Flumioxazin	355	299	10.4	110	28	Positive
Fluometuron	233	72	9.3	105	17	Positive
Fluometuron	233	46	9.3	105	17	Positive
Fluometuron2	233	72	10	105	17	Positive
Fluometuron2	233	46	10	105	17	Positive
Fluopicolide	383	173	11.4	110	24	Positive
Fluopicolide	383	145	11.4	110	45	Positive
Fluoxastrobin	459	427	11.9	140	17	Positive
Fluoxastrobin	459	188	11.9	140	37	Positive
Fluquinconazole	376	349	11.9	120	16	Positive
Fluquinconazole	376	307	11.9	120	24	Positive
Flusilazole	316	247	12.4	120	17	Positive
Flusilazole	316	165	12.4	120	24	Positive
Flutriafol	302	123	9.7	100	28	Positive
Flutriafol	302	70	9.7	100	17	Positive
Fonofos	247	137	13	120	17	Positive
Fonofos	247	109	13	120	35	Positive
Foramsulfuron	453	182	6.15	120	22	Positive
Foramsulfuron	453	139	6.15	120	45	Positive
Forchlorfenuron	248	129	10	110	16	Positive
Forchlorfenuron	248	93	10	110	37	Positive
Fosthiazate	284	228	9.3	100	5	Positive
Fosthiazate	284	104	9.3	100	22	Positive
Fuberidazol	185	157	8	145	22	Positive
Fuberidazol	185	156	8	145	28	Positive
Furalaxyl	302	242	10.8	110	12	Positive
Furalaxyl	302	95	10.8	110	27	Positive
Furathiocarb	383	252	14.3	110	5	Positive
Furathiocarb	383	195	14.3	110	17	Positive
Halofenozide	329	121	11	105	16	Negative
Halofenozide	329	76.9	11	105	36	Negative
Halosulfuron-methyl	434.7	182	7.5	110	17	Positive

Halosulfuron-methyl	434.7	139	7.5	110	45	Positive
Hexaconazole	314	159	13.2	95	28	Positive
Hexaconazole	314	70	13.2	95	17	Positive
Hexaflumuron	461	158	13.9	120	17	Positive
Hexaflumuron	461	141	13.9	120	45	Positive
Hexythiazox	353	228	14.8	100	12	Positive
Hexythiazox	353	168	14.8	100	24	Positive
HT-2	442	263	9.9	110	12	Positive
HT-2	442	215	9.9	110	12	Positive
HT-2-^{13}C22	464	278	9.9	110	12	Positive
Hydramethylnon	495	323	14.3	200	36	Positive
Hydramethylnon	495	170.9	14.3	200	48	Positive
Imazalil	297	201	10.1	115	17	Positive
Imazalil	297	159	10.1	115	22	Positive
Imidacloprid	256	209	6	83	12	Positive
Imidacloprid	256	175	6	83	17	Positive
Indoxacarb	528	203	13.7	110	45	Positive
Indoxacarb	528	150	13.7	110	22	Positive
Ipconazole	334	125	13.9	115	45	Positive
Ipconazole	334	70	13.9	115	24	Positive
Iprovalicarb	321	203	11.9	83	0	Positive
Iprovalicarb	321	119	11.9	83	22	Positive
Isocarbophos	231	121	10	110	22	Positive
Isocarbophos	231	65	10	110	37	Positive
Isofenphos-methyl	332	231	12.8	145	12	Positive
Isofenphos-methyl	332	121	12.8	145	37	Positive
Isoprothiolane	291	231	11.4	83	8	Positive
Isoprothiolane	291	188.8	11.4	83	22	Positive
Isoxaben	333	165	11.3	110	16	Positive
Isoxaben	333	150	11.3	110	48	Positive
Isoxaflutole	359.8	250.9	7.5	95	22	Positive
Isoxaflutole	359.8	220	7.5	95	35	Positive
Isoxaflutole2	359.8	250.9	9.9	95	22	Positive
Isoxaflutole2	359.8	220	9.9	95	35	Positive
Ivermectin B1a	892.5	551.3	16.8	120	16	Positive
Ivermectin B1a	892.5	307	16.8	120	28	Positive
Ivermectin B1b	861.5	495.8	14.1	120	12	Positive
Ivermectin B1b	861.5	323	14.1	120	60	Positive
Kresoxim-methyl	314	267	12.7	85	0	Positive
Kresoxim-methyl	314	222	12.7	85	12	Positive

Lenacil	235	153	9.7	85	17	Positive
Lenacil	235	136	9.7	85	35	Positive
Linuron	249	182	10.8	110	12	Positive
Linuron	249	160	10.8	110	17	Positive
Lufenuron	510.9	158	14.6	138	22	Positive
Lufenuron	510.9	141	14.6	138	45	Positive
Malaoxon	315.07	127	8.6	85	4	Positive
Malaoxon	315.07	99	8.6	85	22	Positive
Malathion	331	126.9	11.4	83	5	Positive
Malathion	331	99	11.4	83	12	Positive
Mandipropamid	411.9	356	11.3	110	5	Positive
Mandipropamid	411.9	328	11.3	110	12	Positive
Mecarbam	330	227	12	65	0	Positive
Mecarbam	330	97	12	65	45	Positive
Mepanipyrim	224	209	11.9	140	16	Positive
Mepanipyrim	224	106	11.9	140	24	Positive
Mesosulfuron-methyl	504	182	6.5	125	24	Positive
Mesosulfuron-methyl	504	139	6.5	125	45	Positive
Metaflumizone	507	287	14.4	140	22	Positive
Metaflumizone	507	178	14.4	140	22	Positive
Metalaxyl	280	220	9.9	95	12	Positive
Metalaxyl	280	160	9.9	95	22	Positive
Metamitron	203	175	6.3	110	17	Positive
Metamitron	203	104	6.3	110	22	Positive
Metazachlor	278	210	9.7	65	0	Positive
Metazachlor	278	134	9.7	65	17	Positive
Metconazole	320	125	13.2	140	37	Positive
Metconazole	320	70	13.2	140	22	Positive
Methabenzthiazuron	222	165	9.6	100	17	Positive
Methabenzthiazuron	222	150	9.6	100	35	Positive
Methacrifos	241	209	10.3	55	0	Positive
Methacrifos	241	125	10.3	55	28	Positive
Methamidophos	141.9	125	2	85	12	Positive
Methamidophos	141.9	94	2	85	12	Positive
Methidathion	302.9	145	10.2	55	0	Positive
Methidathion	302.9	85	10.2	55	17	Positive
Methiocarb	226	169	11	65	0	Positive
Methiocarb	226	121	11	65	17	Positive
Methomyl	162.9	106	5.1	50	5	Positive

Methomyl	162.9	88	5.1	50	0	Positive
Methoprotryne	272	198	10.8	140	24	Positive
Methoprotryne	272	169.9	10.8	140	28	Positive
Methoxyfenozide	369	313	11.5	85	0	Positive
Methoxyfenozide	369	149	11.5	85	12	Positive
Metobromuron	259	170	9.5	120	17	Positive
Metobromuron	259	148	9.5	120	12	Positive
Metolachlor	284	252	12.2	110	12	Positive
Metolachlor	284	176	12.2	110	22	Positive
Metrafenon	409	226.9	13.4	110	24	Positive
Metrafenon	409	209	13.4	110	12	Positive
Metribuzin	215	187	8.3	120	17	Positive
Metribuzin	215	84	8.3	120	22	Positive
Metsulfuron-methyl	382	199	5.9	100	22	Positive
Metsulfuron-methyl	382	167	5.9	100	17	Positive
Mevinphos1	225	193	6.4	65	0	Positive
Mevinphos1	225	127	6.4	65	12	Positive
Mevinphos2	225	193	7	65	0	Positive
Mevinphos2	225	127	7	65	12	Positive
Mexacarbate	223	166	11.5	110	12	Positive
Mexacarbate	223	151	11.5	110	24	Positive
Molinate	188	126	11.5	100	12	Positive
Molinate	188	83	11.5	100	17	Positive
Monocrotophos	224	193	5.5	65	0	Positive
Monocrotophos	224	127	5.5	65	12	Positive
Moxidectin	640.4	622	16.5	148	12	Positive
Moxidectin	640.4	528	16.5	148	4	Positive
Myclobutanil	289	125	11.6	110	35	Positive
Myclobutanil	289	70	11.6	110	17	Positive
Nicosulfuron	4111	213	5.6	102	12	Positive
Nicosulfuron	4111	182	5.6	105	16	Positive
Nitenpyram	271	225	4.9	95	3	Positive
Nitenpyram	271	56	4.9	95	28	Positive
Novaluron	493	158	14	100	22	Positive
Novaluron	493	141	14	100	45	Positive
Omethoat	214	125	4	83	22	Positive
Omethoat	214	109	4	83	24	Positive
OTA	404	358	9.6	120	12	Positive
OTA	404	239	9.6	120	24	Positive
OTA	404	221	9.6	120	37	Positive
OTA-^{13}C20	424	250	9.6	120	24	Positive

Oxadiazon	345	303	14.6	100	12	Positive
Oxadiazon	345	220	14.6	100	17	Positive
Oxadixyl	279	219	7.7	65	5	Positive
Oxadixyl	279	132	7.7	65	35	Positive
Oxamyl	237	90	4.9	60	0	Positive
Oxamyl	237	72	4.9	60	17	Positive
Oxasulfuron	407	150	6.2	120	17	Positive
Oxasulfuron	407	107	6.2	120	45	Positive
Paclobutrazol	294	125	11.4	115	37	Positive
Paclobutrazol	294	70	11.4	115	22	Positive
Penconazole	284	159	12.7	65	28	Positive
Penconazole	284	70	12.7	65	17	Positive
Pencycuron	329	218	13.5	120	12	Positive
Pencycuron	329	125	13.5	120	24	Positive
Pendimethalin	282	212	14.9	85	5	Positive
Pendimethalin	282	194	14.9	85	17	Positive
Permethrin	391	355	10	120	6	Positive
Permethrin	391	183	10	120	12	Positive
Phenmedipham	318	168	10.4	100	4	Positive
Phenmedipham	318	136	10.4	100	22	Positive
Phenthoate	321	247	12.6	75	4	Positive
Phenthoate	321	79	12.6	75	48	Positive
Phosalone	368	182	13.3	65	12	Positive
Phosalone	368	111	13.3	65	45	Positive
Phosmet	317.9	160	10.5	65	12	Positive
Phosmet	317.9	133	10.5	65	37	Positive
Phosphamidon	300	174	7.8	110	8	Positive
Phosphamidon	300	127	7.8	110	16	Positive
Phoxim	299	129	13.2	65	4	Positive
Phoxim	299	77	13.2	65	24	Positive
Picolinafen	377	359	14.4	120	24	Positive
Picolinafen	377	238	14.4	120	32	Positive
Picoxystrobin	368	205	12.5	65	0	Positive
Picoxystrobin	368	145	12.5	65	22	Positive
Piperonylbutoxide	356	177	14.6	120	13	Positive
Piperonylbutoxide	356	119	14.6	120	47	Positive
Pirimicarb	239	182	9.4	110	12	Positive
Pirimicarb	239	72	9.4	110	22	Positive
Pirimiphos-ethyl	334	198	14.6	120	38	Positive
Pirimiphos-ethyl	334	182	14.6	120	42	Positive
Pirimiphos-methyl	306	164	13.4	140	22	Positive

Pirimiphos-methyl	306	108	13.4	140	28	Positive
Prochloraz	376	308	13.3	65	5	Positive
Prochloraz	376	266	13.3	65	12	Positive
Procymidon	301	284	12	65	8	Positive
Procymidon	301	256	12	65	24	Positive
Procymidon	284	255.8	12	110	12	Positive
Procymidon	284	67	12	110	28	Positive
Profenofos	374.9	347	14.2	120	5	Positive
Profenofos	374.9	304.9	14.2	120	17	Positive
Promecarb	208	151	11.2	83	0	Positive
Promecarb	208	109	11.2	83	12	Positive
Prometon	226	184	8.9	110	16	Positive
Prometon	226	142	8.9	110	24	Positive
Propamocarb	189	144	4.4	100	5	Positive
Propamocarb	189	102	4.4	100	17	Positive
Propaquizafop	444	371	14.5	125	12	Positive
Propaquizafop	444	100	14.5	125	17	Positive
Propargit	368	231	15.1	83	5	Positive
Propargit	368	175	15.1	83	12	Positive
Propetamophos	282	156	11.6	125	12	Positive
Propetamophos	282	138	11.6	125	17	Positive
Propham	180	138	9.6	60	4	Positive
Propham	180	120	9.6	60	12	Positive
Propiconazole	342	158.9	13	115	28	Positive
Propiconazole	342	69	13	115	17	Positive
Propoxur	210	168	8.3	55	0	Positive
Propoxur	210	111	8.3	55	12	Positive
Propyzamid	256	190	11.4	105	12	Positive
Propyzamid	256	173	11.4	105	22	Positive
Proquinazid	373	331	15.6	120	5	Positive
Proquinazid	373	289	15.6	120	22	Positive
Prosulfocarb	252	128	14	100	5	Positive
Prosulfocarb	252	91	14	100	22	Positive
Pymetrozin	218	105	5.4	110	22	Positive
Pymetrozin	218	78	5.4	110	45	Positive
Pyracarbolid	218	125	8.6	115	16	Positive
Pyracarbolid	218	96.9	8.6	115	28	Positive
Pyraclostrobin	388	194	13.2	95	5	Positive
Pyraclostrobin	388	163	13.2	95	22	Positive
Pyridaben	365	309	15.7	83	12	Positive
Pyridaben	365	147	15.7	83	24	Positive

Pyridat	379	350.8	16.1	110	4	Positive
Pyridat	379	207	16.1	110	12	Positive
Pyrimethanil	200	107	11	120	22	Positive
Pyrimethanil	200	82	11	120	24	Positive
Pyriproxyfen	322	185	14.8	110	22	Positive
Pyriproxyfen	322	96	14.8	110	12	Positive
Quinalphos	299	163	12.6	100	22	Positive
Quinalphos	299	147	12.6	100	22	Positive
Quinmerac	222	204	5.3	100	17	Positive
Quinmerac	222	141	5.3	100	35	Positive
Quinoclamin	208	88.9	8	125	44	Positive
Quinoclamin	208	76.9	8	125	44	Positive
Quinoxyfen	308	197	14.8	115	35	Positive
Quinoxyfen	308	162	14.8	115	45	Positive
Rimsulfuron	432	325	5.9	110	12	Positive
Rimsulfuron	432	182	5.9	110	22	Positive
Rotenone	395	213	12.4	145	22	Positive
Rotenone	395	192	12.4	145	22	Positive
Scopolamine	304	156	7.2	120	17	Positive
Scopolamine	304	137.9	7.2	120	23	Positive
Scopolamine-^{13}C1-d3	308	160	7.2	120	17	Positive
Scopolamine-^{13}C1-d3	308	142	7.2	120	23	Positive
Secbumeton	226	170	10.5	110	16	Positive
Secbumeton	226	67.9	10.5	110	50	Positive
Silthiopham	268	252	12.6	135	5	Positive
Silthiopham	268	139	12.6	135	17	Positive
Spinosyn A	732.4	142	16.3	155	28	Positive
Spinosyn A	732.4	98	16.3	155	45	Positive
Spinosyn D	746.5	142	16.7	145	35	Positive
Spinosyn D	746.5	98	16.7	145	55	Positive
Spirodiclofen	411	313	15.4	110	5	Positive
Spirodiclofen	411	71	15.4	110	17	Positive
Spiromesifen	388	273	15	110	12	Positive
Spiromesifen	388	255	15	110	24	Positive
Spirotetramat	374	330.3	11.9	120	12	Positive
Spirotetramat	374	302	11.9	120	12	Positive
Spiroxamine	298	144	14.9	125	17	Positive
Spiroxamine	298	100	14.9	125	35	Positive
Sulfentrazone	404	306.9	8.3	110	28	Positive

Sulfentrazone	404	273	8.3	110	36	Positive
Sulfotep	323	115	12.8	120	31	Positive
Sulfotep	323	97	12.8	120	48	Positive
T-2	484.3	305	10.8	110	12	Positive
T-2	484.3	215	10.8	110	24	Positive
T-2	484.3	185	10.8	110	22	Positive
T-2-^{13}C24	508.3	322	10.8	110	12	Positive
TEA	196	139	4.3	110	26	Negative
TEA	196	112	4.3	110	32	Negative
TEA	196	83	4.3	110	32	Negative
TEA-^{13}C2	198	141	4.3	110	26	Negative
Tebuconazole	308	125	12.9	110	37	Positive
Tebuconazole	308	70	12.9	110	22	Positive
Tebufenozid	353	297	12.5	95	0	Positive
Tebufenozid	353	133	12.5	95	17	Positive
Tebufenpyrad	334	145	14.4	145	24	Positive
Tebufenpyrad	334	117	14.4	145	37	Positive
Tebuthiuron	229	172	8.6	105	12	Positive
Tebuthiuron	229	116	8.6	105	24	Positive
Teflubenzuron	379	359	14.6	110	0	Negative
Teflubenzuron	379	339	14.6	110	4	Negative
Temephos	467	419	14.5	155	22	Positive
Temephos	467	124.9	14.5	155	44	Positive
Tepraloxydim1	342	250	6.45	140	12	Positive
Tepraloxydim1	342	166	6.45	140	22	Positive
Tepraloxydim2	342	250	7.5	140	12	Positive
Tepraloxydim2	342	166	7.5	140	22	Positive
Terbufos	289	232.9	14.5	110	0	Positive
Terbufos	289	57	14.5	110	16	Positive
Tetrachlorvinphos	367	206	12.6	120	39	Positive
Tetrachlorvinphos	367	127	12.6	120	48	Positive
Tetraconazole	372	159	12.1	140	28	Positive
Tetraconazole	372	70	12.1	140	22	Positive
Tetrametrin	349	164	14.2	120	35	Positive
Tetrametrin	349	107	14.2	120	50	Positive
Thiabendazol	202	175	7.7	140	24	Positive
Thiabendazol	202	131	7.7	140	35	Positive
Thiacloprid	253	126	6.95	110	22	Positive
Thiacloprid	253	90	6.95	110	37	Positive
Thiamethoxam	292	211	5.3	85	5	Positive
Thiamethoxam	292	181	5.3	85	22	Positive

Thidiazuron	221	101.9	8.4	107	16	Positive
Thidiazuron	221	77	8.4	107	50	Positive
Thifensulfuron-methyl	388	205	5.9	115	24	Positive
Thifensulfuron-methyl	388	167	5.9	115	12	Positive
Thiodicarb	355	108	9.2	82	12	Positive
Thiodicarb	355	88	9.2	82	12	Positive
Thiofanox	241	184	9.3	110	5	Positive
Thiofanox	241	57	9.3	110	17	Positive
Tolclofos-methyl	301	269	13.4	115	12	Positive
Tolclofos-methyl	301	125	13.4	115	17	Positive
Tolylfluanide	346.9	238	12	65	0	Positive
Tolylfluanide	346.9	137	12	65	24	Positive
Tralkoxydim	330	284	16.4	165	5	Positive
Tralkoxydim	330	138	16.4	165	17	Positive
Tralkoxydim	330	96	16.4	165	24	Positive
Triadimefon	294	197	11.5	100	12	Positive
Triadimefon	294	69	11.5	100	22	Positive
Triadimenol	296	99	11.85	65	12	Positive
Triadimenol	296	70	11.85	65	5	Positive
Triasulfuron	401.9	167	6.7	140	12	Positive
Triasulfuron	401.9	141	6.7	140	12	Positive
Triazophos	314	162	11.7	110	17	Positive
Triazophos	314	119	11.7	110	35	Positive
Tribenuron-methyl	396	181	6.4	110	17	Positive
Tribenuron-methyl	396	155	6.4	110	5	Positive
Trichlorfon	256.9	221	6.4	83	5	Positive
Trichlorfon	256.9	109	6.4	83	17	Positive
Tricyclazol	190	163	7.2	140	22	Positive
Tricyclazol	190	136	7.2	140	28	Positive
Trietazin	230	202	11.9	105	17	Positive
Trietazin	230	99	11.9	105	24	Positive
Trifloxystrobin	409	186	13.8	110	12	Positive
Trifloxystrobin	409	145	13.8	110	45	Positive
Triflumizol	346	278	13.9	85	5	Positive
Triflumizol	346	73	13.9	85	12	Positive
Triflumuron	359	156	13.3	100	12	Positive
Triflumuron	359	139	13.3	100	35	Positive
Trimethacarb	194	137	9.9	83	4	Positive
Trimethacarb	194	122	9.9	83	28	Positive

Triticonazole	318	125	12	100	37	Positive
Triticonazole	318	70	12	100	12	Positive
Uniconazole-P	292	125	12.4	135	36	Positive
Uniconazole-P	292	70	12.4	135	24	Positive
Vamidothion	288	146	6.4	95	8	Positive
Vamidothion	288	58	6.4	95	50	Positive
ZON	317	175	11.5	110	24	Negative
ZON	317	130.8	11.5	110	33	Negative
ZON-^{13}C18	335	185	11.5	110	24	Negative
Zoxamide	336	187	13	120	22	Positive
Zoxamide	336	159	13	120	45	Positive

AME: alternariol monomethyl ether, AOH: alternariol, DON: deoxynivalenol, FB1: fumonisin B1, FB2: fumonisin B2, FB3: fumonisin B3, OTA: ochratoin A, TEA: tenuazonic acid, ZON: zearalenone
